# Optimization of Culturomics Strategy in Human Fecal Samples

**DOI:** 10.3389/fmicb.2019.02891

**Published:** 2019-12-17

**Authors:** Yuxiao Chang, Fengyi Hou, Zhiyuan Pan, Zongyu Huang, Ni Han, Lei Bin, Huimin Deng, Zhengchao Li, Lei Ding, Hong Gao, Fachao Zhi, Ruifu Yang, Yujing Bi

**Affiliations:** ^1^State Key Laboratory of Pathogen and Biosecurity, Beijing Institute of Microbiology and Epidemiology, Beijing, China; ^2^Guangdong Provincial Key Laboratory of Gastroenterology, Institute of Gastroenterology of Guangdong Province, Department of Gastroenterology, Nanfang Hospital, Southern Medical University, Guangzhou, China; ^3^Beijing Shijitan Hospital, Capital Medical University, Beijing, China

**Keywords:** culturomics, gut microbiota, optimization strategy, medium supplementation, colony picking

## Abstract

Most bacteria in the human gut are difficult to culture, and culturomics has been designed to overcome this issue. Culturomics makes it possible to obtain living bacteria for further experiments, unlike metagenomics. However, culturomics is work-intensive, which prevents its wide application. In this study, we performed a 30-day continuous enrichment in blood culture bottles and cultured bacterial isolates from pre-cultures removed at different time points. We compared the bacteria isolated from the enriched culture with or without adding fresh medium after each pre-culture was removed. We also compared “experienced” colony picking (i.e., picking two to three colonies for each recognized colony type) and picking all the colonies from each plate. In total, from five fecal samples, 106 species were isolated, including three novel species and six that have not previously been isolated from the human body. Adding fresh medium to the culture increased the rate of bacterial species isolation by 22% compared with the non-supplemented culture. Picking all colonies increased the rate of bacterial isolation by only 8.5% compared with experienced colony picking. After optimization through statistical analysis and simulation, sampling aerobic and anaerobic enrichment cultures at six and seven time-points, respectively, is likely to isolate >90% of bacterial species, reducing the workload by 40%. In conclusion, an extended enrichment step ensures isolation of different bacterial species at different time-points, while adding the same quantity of fresh medium after sampling, the experienced picking and the optimized time-points favor the chance of isolating more bacterial species with less work.

## Introduction

The human intestine provides a favorable habitat for microorganisms. The adult intestine can contain as many as 10^14^ bacteria, which is close to 1.3 times the number of cells in the human body (Sender et al., 2016). The gut microbiota performs metabolic functions that the human body is unable to achieve by itself. Many studies have shown that microorganisms in the intestine are not only closely associated with diseases of the digestive tract (Rivière et al., 2016; Ming et al., 2017), but also play an extremely important role in nervous system diseases, metabolic diseases, and cardiovascular diseases (Yarandi et al., 2016; Liu et al., 2017, 2018; Li et al., 2019). Therefore, it is important to identify the bacteria that reside in the intestines. While metagenomics has revolutionized the study of the human microbiota and our understanding of gut microbes, health, and disease (Lagier et al., 2016), it has some disadvantages, such as a high detection threshold and DNA extraction errors. Standard culturing approaches can compensate for these deficiencies (Lagier et al., 2012a). To study the relationship between bacteria and health or disease at the strain level, it is necessary to maintain living bacteria *in vitro*. Thus, culturing is a key step in deciphering the role of specific microorganisms and microbial communities in host health. Given the increasing number of known microorganisms, it is becoming more and more difficult to discover new types of microorganisms. Only 1525 bacteria can be detected using today’s technologies, and 80% of bacteria are considered to be unculturable (Lagier et al., 2018). Therefore, more effective culture methods and conditions are needed to isolate and culture more bacteria.

Lagier et al. (2016, 2015) summarized 18 optimal culture conditions and identified blood culture bottles, rumen fluid, and sheep’s blood as three key nutrient substrates for growing bacteria. This group also attempted to isolate many unknown bacteria from the intestine by extending the pre-incubation time. Our study is based on this method of prolonging the pre-incubation step and using blood culture bottles. In contrast, however, we extracted samples from the pre-cultures at different time points, supplemented the culture with an equal volume of fresh medium, and investigated the effect of this approach. By comparing and analyzing the bacteria isolated at different time-points, we were able to optimize the culturomics conditions to reduce the substantial amount of work that is typically required for this method.

## Materials and Methods

### Samples

Fresh fecal samples were collected from five healthy people (the samples were numbered F1–F5). The inclusion criteria for these volunteers were: (1) 25-35-year-old, non-pregnant, healthy adults with a healthy lifestyle and good eating habits; (2) in the past 6 months, had not taken antibacterial drugs and other drugs that interfere with the intestinal micro-ecology; (3) no smoking, alcohol use, or other unhealthy habits; (4) no gastrointestinal tumors, polyps, Integrated brand dealer (IBD), or other digestive diseases; (5) no major diseases such as diabetes, heart disease, high blood pressure, or malignant tumors. Consent was obtained from each volunteer, and the research was approved by the Ethics Committee of Beijing Shijitan Hospital (Beijing, China; agreement no. 2018KY55). The fresh stool samples were used immediately after collection, without being stored for any length of time. Fresh feces were collected by the fecal collector (Lianghua health, Beijing, China), then suspended in 10 ml of PBS per 1 g of feces. The suspension was reserved for further experiments.

### Prolonged Pre-incubation

The stool suspension was pre-incubated in blood culture bottles (Autobio, Zhengzhou, China) supplemented with 10% sheep blood and 10% rumen fluid (ELITE-MEDIA, Shanghai, China). Half of the blood culture bottles were incubated under anaerobic conditions, and the other half were incubated under aerobic conditions. All anaerobic conditions are cultured in an anaerobic incubator (Ruskinn, United Kingdom), including pre-cultured or enrichment culture under anaerobic conditions, and the solid plate and liquid nutrient medium of YCFA is put into the anaerobic incubator 2 days in advance, and then cultured in the same anaerobic incubator. Each sample was isolated and cultured independently. The gas in the anaerobic tank is composed of 80% nitrogen, 10% carbon dioxide, and 10% hydrogen to ensure the anaerobic environment. Given the prolonged culture time, samples were periodically removed from the blood culture bottle for further cultivation. Every third day, from day 0 to day 30 3 ml of the pre-cultures was extracted and cultivated. In total, 11 samples were extracted from each pre-incubation culture. In the supplementation group, an equal volume of fresh medium was added to the original culture after each sample was taken. The samples were plated in duplicate to YCFA agar and incubated at 37°C, aerobically or anaerobically.

### Colony Picking Strategies

We selected plates containing 100–1000 colonies and picked colonies according to one of two methods. The first method was to pick different morphology clones (based on color, shape, and size), then for the same morphology additional 1–2 colonies were picked, and averagely about 10–15 colonies were selected per plate. This method is referred to hereafter as “experienced colony picking.” The second method was to pick all colonies on the plate, which is referred to hereafter as “picking all.”

### Detection by MALDI-TOF Mass Spectrometry

MALDI-TOF Mass Spectrometry was used to identify the bacteria. Each colony was evenly applied to the MALDI-TOF MS 96 target plate, then covered with lysis solution (70% formic acid) and a matrix solution (a saturated solution of α-cyano-4-hydroxycinnamic acid in 50% acetonitrile). Next, the target plate was detected using an Autof MS1000 spectrometer (Autobio, Zhengzhou, China). If the colony’s spectrum had a score ≥9.0, it was considered to be correctly identified at the species level.

### 16S rRNA Gene Sequencing

If the colonies could not be accurately identified by MALDI-TOF, then the isolate was identified by 16S rRNA gene sequencing. Total DNA was extracted from the isolate using a Bacterial DNA Kit (Qiagen, German), and specific primers (27F: 5′-AGAGTTTGATCCTGGCTCAG-3′; 1492R: 5′-GGTTACCTTGTTACGACTT-3′) were used to detect the conserved 16S V4 region. If the 16S rRNA gene sequence exhibited less than 98.65% similarity to that of known species, it was considered to be a potentially new species.

### pH Measurement

The pH of each pre-culture at different time-points was measured using a pH meter (METTLER TOLEDO Seven2GO S2, Shanghai, China). Before measuring, the instrument probe was rinsed with distilled water at a neutral pH, after which the pH instrument was calibrated using the two-point method (pH = 4.01 and pH = 6.86). Finally, the pH value of the bacterial cultures was measured.

### Statistical Analysis

#### Generating a Best-Fit Equation Based on the Number of Bacterial Species Identified Depending on the Number of Samples Taken

We counted the species of bacteria identified in the supplementation group (S) and the non supplementation group (NS). There were 120 possible combinations when the five fecal samples were taken in random order. We calculated the number of bacterial species identified after one sample was taken for all possible combinations, then calculated the mean of the 120 combinations. Finally, we used Microsoft Excel software to fit the power function and calculated the number of bacterial species identified when 6–10 samples were taken. If the sample number is set to *x*_*1*_, and the species of bacteria in the non supplementation group (NS) and all groups (S + NS) are set to *f*_*0*_ and *f*_*1*_, respectively, then the number of bacterial species is calculated as follows:

$$\left\{ \begin{array}{c} f_{0}\left(x_{1}\right)=33.302x_{1}^{0.609}\left(R^{2}=1\right)\\ f_{1}\left(x_{1}\right)=44.396x_{1}^{0.553}\left(R^{2}=1\right) \end{array}\right.$$

#### Multiple Linear Regression Analysis of the Presence of Bacteria and the pH

First, we counted the number of bacterial species identified after each sample was taken from each culture condition (for five samples total), and measured the pH of the culture each time a sample was taken. Then we assigned a value of 1 to indicate the presence of bacteria, or a value of 0 to indicate the absence of bacteria. Finally, we performed multiple linear regression analysis of bacterial presence or absence and the pH value (with *p* < 0.05 considered to be significant). When the partial regression coefficient was positive, the pH value tended to be higher, so the bacteria associated with a positive partial regression coefficient were considered to be alkaline-related bacteria, and those associated with a negative partial regression coefficient were considered to be acid-related bacteria.

#### Calculating the Optimal Sampling Rate

If *n* is the number of times that samples are taken from the culture (*n* = 1–11), then there are $\text{b}=C_{11}^{n}$combinations when we take *n* samples; the number of combinations is 1-*i* (*i* = b), and the number of stool sample is a (a = 1–5). For sample a, the number of bacterial species present in group i after taking n samples is*N*_*a,n,i*_, so the average extraction rate of for a samples taken n times is

$$p_{a,n}=\bar{\sum_{\text{i}=1}^{b}\frac{N_{a,n,i}}{N_{a,11,1}}}$$

and the average extraction rate for five samples is

$$P_{n}=\bar{p_{a,n}}$$

The above calculation was performed using Python software.

#### Generating a Best-Fit Equation for the Number of Bacterial Species Identified Depending on the Workload Before and After Optimization of the Method

If we define taking a sample from aerobic and anaerobic pre-cultures as 1 workload, then the workload of taking one fecal sample prior to optimization is 11. By fitting the number of bacterial species identified to varying numbers of samples taken, we can calculate the number of bacterial species identified *f*_*2*_ based on the original (non-optimized) workload *x*_*2*_:

$$\left\{ \begin{array}{c} x_{2}=11\,x_{1}\\ f_{2}\,\left(x_{2}\right)=f_{1}\,\left(x_{1}\right) \end{array}\right.\to f_{2}\,\left(x_{2}\right)=11.783\,x_{2}^{0.553}$$

The optimized scheme is that separating two samples at a time through the newly optimized selection time, the total workload was 13 each time two samples were taken, and 91% of the bacterial species identified using the non-optimized method were identified using the optimized method. We can calculate the number of bacterial species identified *f*_*3*_ based on the workload *x*_*3*_ after optimization:

$$\left\{ \begin{array}{c} x_{3}=\frac{13}{2}\,x_{1}\\ f_{3}\,\left(x_{3}\right)=91\%\,f_{1}\,\left(x_{1}\right) \end{array}\right.\to f_{3}\,\left(x_{3}\right)=14.344\,x_{3}^{0.553}$$

## Results

### Bacteria Isolated From Five Samples

**FIGURE 1:**
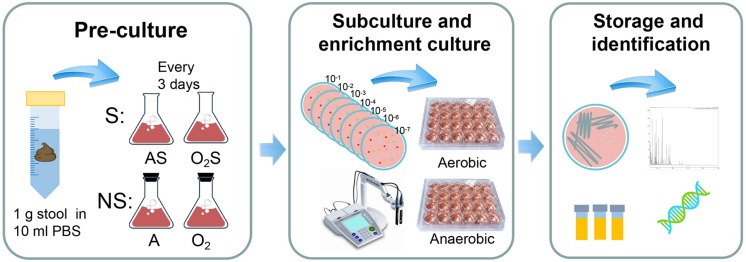
Experimental flow chart. The experiment was divided into three steps. The first step is pre-incubation: 1 g of each stool sample was suspended in 10 ml of PBS and allowed to stand for 5 min, after which 0.5 ml of the suspension was added to blood culture bottles containing sheep’s blood and rumen fluid. The bottles were divided into two groups: the supplemented group (S) and non-supplemented group (NS). The supplemented group was then further divided into the aerobic supplemented group (O_2_S) and the anaerobic supplemented group (AS). Similarly, the non-supplemented group was divided into the aerobic non-supplemented group (O_2_) and the anaerobic non-supplemented group (A). The second step was subculturing and enrichment: samples were taken from the pre-cultures every 3 days and subcultured aerobically and anaerobically on solid YCFA media, and the pH of the pre-cultures was measured using a pH meter. When colonies appeared on the plates, individual colonies were picked and transferred to liquid culture enrichment medium (YCFA) in 24-well plates. The last step was bacterial storage and identification: the enrichment cultures were frozen in glycerol and inoculated to solid medium at the same time. Then, the colonies were tested by MALDI-TOF mass spectrometry. Unidentified colonies were evaluated by 16S rRNA gene sequencing. All bacteria were incubated aerobically for 1 day or anaerobically for 3 days at 37°C.

The experiment protocol is shown in Figure 1. Briefly, stool suspensions were pre-cultured under different conditions: anaerobically with supplemented medium (AS); aerobically with supplemented medium (O_2_S); anaerobically with non-supplemented medium (A); or aerobically with non-supplemented medium (O_2_). Every three days samples were taken from the pre-cultures and subcultured on YCFA agar (Duncan et al., 2002; Browne et al., 2016). The colonies were identified by MALDI-TOF or 16S rRNA gene sequencing.

**FIGURE 2:**
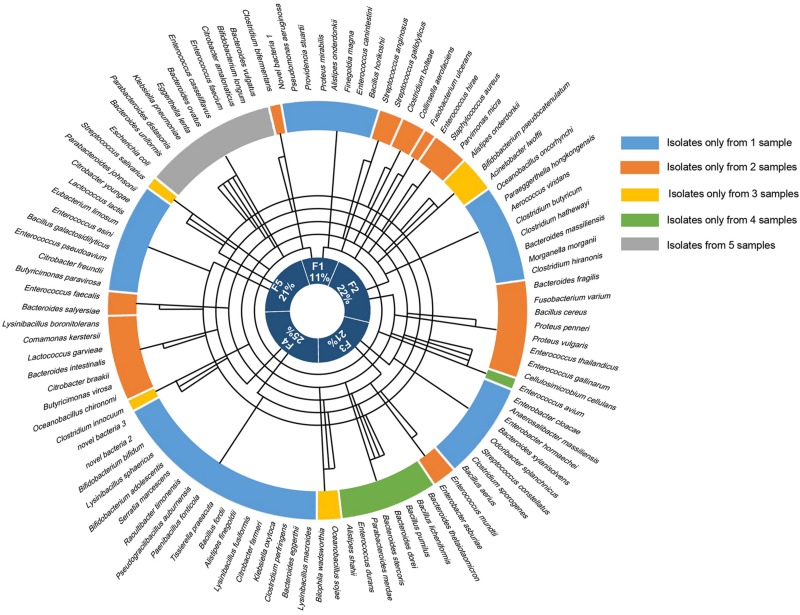
The bacteria identified from each of the fecal samples. The outermost circle shows all 106 of the bacteria that were identified, the five colors in the next circle represent the fecal samples that the bacteria were isolated from, and the innermost circle shows the proportion of bacterial species that were isolated from each of the five samples. The line in the middle connects the sample to the bacterial species that were isolated from that sample.

Altogether, the bacteria isolated from the five samples belonged to 1 kingdom, 5 phyla, 10 classes, 14 orders, 25 families, 41 genera, and 106 species (Figure 2), and totally 1718 colonies were isolated, the numbers of colonies analyzed for each condition is shown in Supplementary Table S1. The horizontal composition of each sample at the genus level was analyzed (Supplementary Figure S1A). Bacteria isolated from only one of the fecal samples accounted for 48% of the total bacterial species identified (Supplementary Figure S1B), meaning that nearly half of the bacteria were isolated from a single sample. Further analysis of these bacteria revealed the presence of three novel species which had less than 98.65% similarity with other species, based on 16S rRNA gene sequencing (Abdul-Hussein and Atia, 2016), the results of 16S rRNA blast are shown in Table 1. Comparison to the intestinal bacteria library established by Bilen et al. (2018) and to the literature identified two strains as having never been isolated from the human intestine and six strains as having never been isolated from the human body (Table 2).

**TABLE 1:** Characteristics of the three novel bacterial species.

	**Name**	**Sample source**	**Group**	**Closest relatives**	**Identity**
New species	Novel bacteria 1	F1	S	*Longicatena caecimuris*	93.15%
	Novel bacteria 2	F4	NS	*Bacillus alcalophilus*	96.11%
	Novel bacteria 3	F4	S	*Pseudogracilibacillus auburnensis*	97.37%

*S, supplemented medium group; NS, non-supplemented medium group.*

**TABLE 2:** Characteristics of eight bacterial species not previously isolated from the human intestine or body.

	**Name**	**Sample source**	**Group**	**Bacterial isolation source**	**References**
Not previously isolated from the gut	*Eggerthella hongkongensis*	F2	NS	Blood from a patient with bacteremia	Lau et al., 2004
	*Fusobacterium ulcerans*	F1 and F3	S and NS	Tropical ulcer tissue	Adriaans and Shah, 1988
Not previously isolated	*Paenibacillus fonticola*	F4	NS	Warm spring	Chou et al., 2007
from the human body	*Bacillus horikoshii*	F1	NS	Puffer fish liver	Lu and Yi, 2009
	*Bacillus aerius*	F3	S and NS	Cryogenic tubes used for collecting air samples from high altitudes	Shivaji et al., 2006
	*Lysinibacillus macroides*	F4	S and NS	Cow dung	Coorevits et al., 2011
	*Pseudogracilibacillus auburnensis*	F4	S and NS	*Zea mays* rhizosphere	Glaeser et al., 2014
	*Oceanobacillus chironomi*	F4 and F5	S and NS	Chironomid egg mass	Raats and Halpern, 2007

*S, supplemented medium group; NS, non-supplemented medium group.*

### Comparison of Bacterial Isolation From the Supplemented and Non-supplemented Groups

**FIGURE 3:**
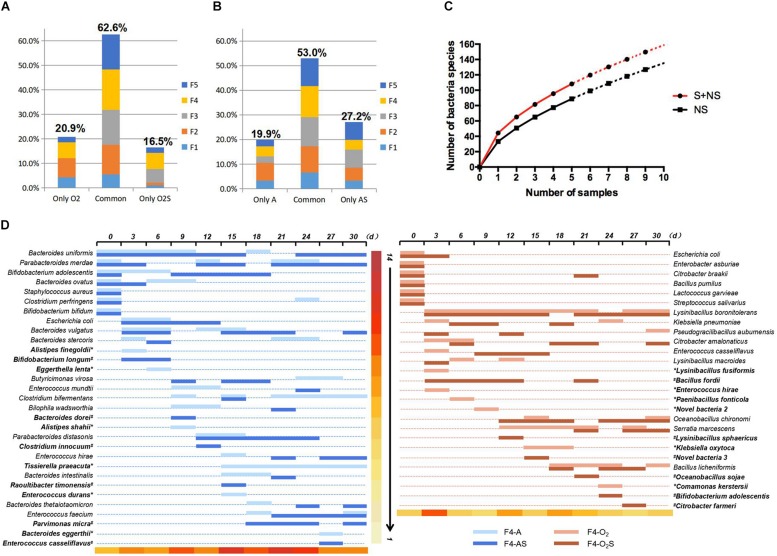
**(A)** Bacteria isolated only from the aerobic supplemented group, only from the aerobic non-supplemented group, and from both groups. **(B)** Bacteria isolated only from the anaerobic supplemented group, only from the anaerobic non-supplemented group, and from both groups. **(C)** Equation fitted to the number of bacterial species identified depending on the number of samples taken. **(D)** Bacteria isolated from fecal sample 4 over time. The bacteria identified at each time point are represented by horizontal lines of different colors. ^∗^indicates bacteria that were only isolated from the supplemented group, and ^#^indicates bacteria that were only isolated from the non-supplemented group. The color scale at the bottom of the figure indicates the number of bacterial species isolated from the sample taken at each time point.

The bacteria isolated from the supplemented group and the non-supplemented group were compared (Figure 3). Under aerobic conditions (Figure 3A), 16.5% of bacteria were only isolated from the supplemented group (O_2_S), while 20.9% were only isolated from the non-supplemented group (O_2_). The other 62.6% of bacterial strains were isolated from both groups. Similarly, under anaerobic conditions (Figure 3B), 27.2 and 19.9% of the bacterial species were only isolated from the supplemented (AS) or non-supplemented group (A), respectively, while 53% of bacteria were isolated from both groups. Statistical analysis was used to fit an equation to the number of bacterial species identified depending on the number of samples taken (Figure 3C). Based on this equation, we can draw two conclusions: as the number of samples increases, more bacteria will be isolated; and supplementing the medium will lead to the isolation of more bacteria. The details of the bacteria isolated from sample 4 (F4) are shown in Figure 3D, and the details for the other four samples are shown in Supplementary Figures S2A–D. Interestingly, some bacteria appeared to be present throughout the pre-culture period (such as *Bacteroides uniformis* and *Lysinibacillus boronitolerans*), but some species were only present in the early stage, and others appeared later. The bacteria that were only isolated from the supplemented or non-supplemented group are indicated in bold in the figure. We noticed that most of the bacteria that were only isolated from a single condition appeared late in the pre-incubation period. The new species also appeared later in this period (Table 1). This indicates that a prolonged culturing time is needed to isolate more species. Thus, the number of new bacteria that are isolated can be increased by prolonging pre-culture time and adding fresh culture medium each time a sample is taken.

### Bacterial Presence and pH Value

**FIGURE 4:**
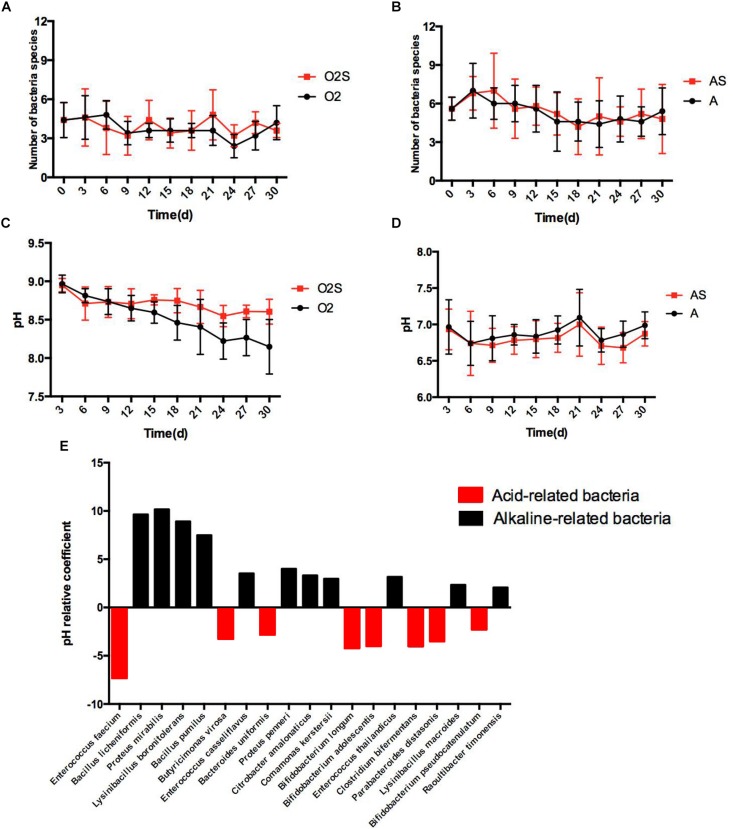
**(A)** The number of bacterial species isolated varies with time under aerobic conditions. **(B)** The number of bacterial species isolated varies with time under anaerobic conditions. **(C)** The pH value of the pre-culture varies with time under aerobic conditions. **(D)** The pH value of the pre-cultures varies with time under anaerobic conditions. Results are shown as means ± SEM. **(E)** The effects of pH on the likelihood of isolating different bacteria, *p* < 0.05.

Different bacteria can dominate the culture at different time-points. The changes in bacterial numbers depending on pre-incubation time are shown in Figures 4A,B, under both aerobic and anaerobic conditions. We next asked whether changes in the pH of the culture caused this change. To assess this, the pH was measured each time a sample was taken from the blood culture bottles (Figures 4C,D), and multiple linear regression analysis of bacterial presence and pH value was performed (Figure 4E). The partial regression coefficient that was obtained was defined as the pH correlation coefficient. The value of the coefficient represents the influence of the pH on the presence of different bacteria: a negative coefficient indicates the presence of acid-related bacteria, and a positive coefficient indicates the presence of alkaline-related bacteria. We found that some bacteria are consistently acid-related (such as *Enterococcus faecium* and *Butyricimonas virosa*), and others are alkaline-related (such as *Proteus mirabilis* and *Bacillus pumilus*). This finding indicates that different habitats are critical for the growth of different types of bacteria.

### Optimization of the Culturomics Strategy

**FIGURE 5:**
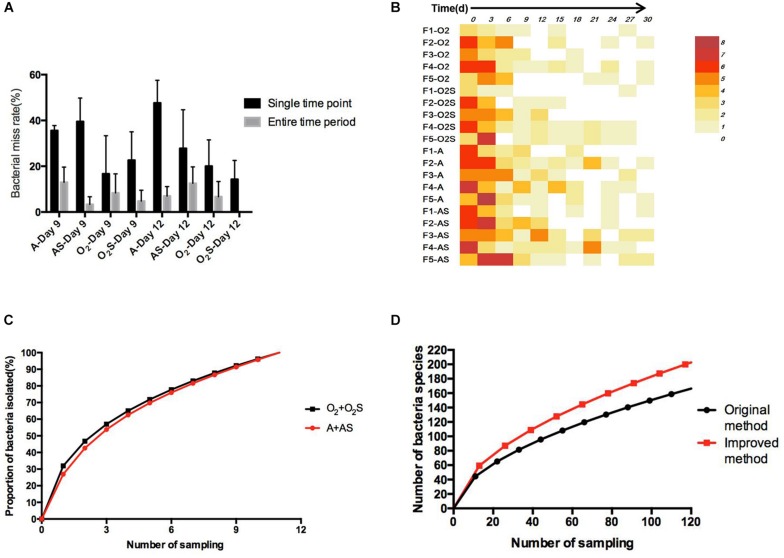
**(A)** The proportion of bacteria missed in different groups at a single time point and over the entire time period. Results are shown as means ± SEM. **(B)** The number of bacteria not isolated from the sample taken at the previous time point compared with the number of bacteria isolated from the current sample. **(C)** The change in bacterial identification rate over time. **(D)** Equation fitted to the number of bacterial species depending on the workload before and after optimization of the method.

To identify any possible omissions that may have been made due to randomly selecting clones, we first compared two colony-picking strategies: one called “experienced picking” (2–3 colonies per plate were picked according to the color, shape, and size); the other called “picking all” (selecting all the colonies on one plate). Three samples (F1, F4, and F5) and two time-points (Days 9 and 12) were selected to compare the difference between the “experienced picking” and “picking all” methods. Two omission rates were calculated: the single time point loss rate, meaning of the number of bacteria identified using the picking all method minus those identified using the experienced picking method, divided by those identified using the picking all method; and the time period miss rate, meaning the number of bacterial species that were missed at a single time point and not isolated at other time points divided by the number of bacterial species identified using the picking all method (Figure 5A). The average single time point loss rate was 32.9%, and the miss rate for entire time period was 8.5%.

Throughout for the 30-day pre-incubation period, samples were taken at 11 time-points. Different species were identified at different time-points, as shown in Figure 5B, suggesting that prolonged pre-incubation was effective for increasing the number of isolated strains. However, as the incubation time increased, the number of new bacteria identified gradually decreased under both aerobic and anaerobic conditions (Supplementary Figures S3A,B). Therefore, it is not advantageous to prolong the pre-culture time indefinitely, nor is it necessarily useful to take samples at each time point tested here. Thus, we evaluated the best time points for picking colonies during this period. The bacterial identification efficiency was defined as the number of bacterial species isolated at n time points divided by the total number of bacterial species isolated at 11 time-points (*n* = 0–11). We fitted an equation to the relationship between bacterial identification efficiency and the number of extraction time-points (n) (Figure 5C). When *n* = 6, the average bacterial identification efficiency was approximately 80%. Under aerobic or anaerobic conditions, the highest identification rate was observed when *n* = 5, 6, or 7 (Tables 3, 4). It seemed reasonable to include an extra sampling time point beyond that identified as yielding the highest identification rate, so we selected seven time points and six time-points as the optimal sampling rate for cultures grown under anaerobic and aerobic conditions, respectively, and found that the identification efficiency at these time-points was greater than 90%. Based on these results, we optimized the bacterial isolation method based on the newly optimized sampling times by fitting an equation to the number of bacteria identified compared to the workload required, before and after optimization of the method (Figure 5D). The optimized scheme is that separating two samples at a time through the newly optimized selection time.

**TABLE 3:** Bacterial isolation rate under aerobic conditions.

**Number of**	**Days on which**	**Bacterial isolation**
**samples taken**	**samples were taken**	**rate (%)**
5	(Day ‘0,’ ‘3,’ ‘6,’ ‘9,’ ‘27’)	80.90
	(Day ‘0,’ ‘3,’ ‘6,’ ‘12,’ ‘27’)	79.23
	(Day ‘0,’ ‘3,’ ‘9,’ ‘18,’ ‘27’)	79.12
6	(Day ‘0,’ ‘3,’ ‘6,’ ‘9,’ ‘15,’ ‘27’)	86.57
	(Day ‘0,’ ‘3,’ ‘6,’ ‘9,’ ‘18,’ ‘27’)	86.53
	(Day ‘0,’ ‘3,’ ‘6,’ ‘9,’ ‘12,’ ‘27’)	86.00
7	(Day ‘0,’ ‘3,’ ‘6,’ ‘9,’ ‘15,’ ‘27,’ ‘30’)	91.35
	(Day ‘0,’ ‘3,’ ‘6,’ ‘9,’ ‘12,’ ‘27,’ ‘30’)	90.78
	(Day ‘0,’ ‘3,’ ‘6,’ ‘9,’ ‘12,’ ‘15,’ ‘27’)	90.48

**TABLE 4:** Bacterial isolation rate under anaerobic conditions.

**Number of**	**Days on which**	**Bacterial isolation**
**samples taken**	**samples were taken**	**rate (%)**
5	(Day ‘0,’ ‘3,’ ‘6,’ ‘15,’ ‘27’)	89.89
	(Day ‘0,’ ‘3,’ ‘6,’ ‘18,’ ‘27’)	88.33
	(Day ‘0,’ ‘3,’ ‘6,’ ‘9,’ ‘27’)	88.15
6	(Day ‘0,’ ‘3,’ ‘6,’ ‘9,’ ‘15,’ ‘27’)	92.63
	(Day ‘0,’ ‘3,’ ‘6,’ ‘15,’ ‘24,’ ‘27’)	92.55
	(Day ‘0,’ ‘3,’ ‘6,’ ‘15,’ ‘18,’ ‘27’)	92.07
7	(Day ‘0,’ ‘3,’ ‘6,’ ‘9,’ ‘15,’ ‘24,’ ‘27’)	95.29
	(Day ‘0,’ ‘3,’ ‘6,’ ‘9,’ ‘15,’ ‘18,’ ‘27’)	94.81
	(Day ‘0,’ ‘3,’ ‘6,’ ‘9,’ ‘15,’ ‘21,’ ‘27’)	94.55

## Discussion

Culturomics is a culture method that uses a variety of culture conditions, MALDI-TOF mass spectrometry, and 16S rRNA gene sequencing to identify bacterial species, intending to isolate as many bacteria as possible (Lagier et al., 2018). Successfully isolating living bacteria is crucial for being able to carry out subsequent experimental work, and many studies of probiotics or harmful bacteria have benefited from the isolation of bacterial strains (Kostic et al., 2013; Mathilde et al., 2014; Lenoir et al., 2016; Ottman et al., 2016; Yang et al., 2017). Culturomics is not only used for the study of intestinal microorganisms but has also been used to investigate microorganisms in the urinary system, vagina, mouth, and breast milk (Wein et al., 2015; Fenollar and Raoult, 2016; Si et al., 2017; Iszatt et al., 2019). Nevertheless, the optimal culture conditions for many bacteria (such as *Proteus*, *Microaerobacteria*, and *Halophilicus*) are very specific and need to be tested one by one, which multiplies the workload needed to identify the appropriate conditions. Therefore, in addition to culturing conditions, optimization of the experimental strategy is also an important aspect of culturomics. In this study, we assessed multiple culturing strategies and developed an optimized protocol designed to reduce the overall workload.

It has been reported that increasing the concentration of lipids, vitamins, and fecal extract and applying heat shock, active filtration, and passive filtration are conducive to isolating more bacteria (Eeward and Fitzgerald, 1951; Eddy and Ingram, 1953; Hecker et al., 1996; Goodman et al., 2011; Lagier et al., 2012b). Lagier et al. tested 212 culturing conditions and identified 18 optimal conditions for identifying unknown bacteria, such as adding sheep’s blood and rumen fluid to the blood culture bottle and prolonging the pre-incubation time. However, as the culturing period progresses, the concentration of nutrients in the culture decreases, which is not conducive to the growth of some bacteria. Therefore, we tested the effect of regularly supplementing the culture with fresh media. We found that 22% of bacteria were only isolated from the supplemented group, indicating that supplementation with fresh media during the pre-culture period strongly promotes the isolation of more bacteria. Besides, some bacteria were only isolated from the non-supplemented group, which means that the two conditions are complementary and should be used in combination. As indicated by the equation fitted to the number of bacterial species identified depending on the number of samples taken, the number of bacterial species identified from the non-supplemented group increased significantly when a supplemented group was also included.

The decomposition of organic and inorganic salts can cause changes in the pH of the culture medium (Skirvin et al., 1986), which can also affect the growth of some bacteria. Some bacteria grow better at certain pH values, while the growth of others is suppressed at the same value. In this study, we measured the pH of the pre-cultures at each sampling time-point and found that it changed continuously over the course of the pre-incubation period. Multiple linear regression analysis of the presence of bacteria and pH indicated that the optimal pH value for each bacterial species is different. Some bacteria grow well in an acidic environment, such as *E. faecium*, *B. virosa*, and *B. uniformis*, and some bacteria are better suited to alkaline environments, such as *Bacillus licheniformis*, *P. mirabilis*, and *L. boronitolerans*. Therefore, the pH of the culturing microenvironment may affect which bacteria are isolated at different time points. However, pH value is not the only factor determining the differences in bacterial species isolated from different time points, the growth rate or survivability of bacteria may also be one of the reasons. A number of environmental factors, including nutrients, pH values, and temperature, affect the number and metabolic activity of bacteria in a culture, and in some cases results in the production of bacteriocins (O’Shea et al., 2011; Dicks et al., 2018), which may be one explanation for why the isolated bacteria were different at different time points. For example, *Escherichia coli* was isolated from sample 1 taken on day 0 from the aerobic culture but had disappeared by day 6, when *Pseudomonas aeruginosa* was isolated. This could be explained by the fact that *P. aeruginosa* can secrete pyocyanin to inhibit the growth of *E. coli* (Stackebrandt and Ebers, 2006). Therefore, studying the interactions between bacteria and the factors that affect these relationships could help isolate more unknown bacteria.

Culturomics is a time-consuming and labor-intensive process that involves a substantial amount of work. Thus, an important question is how to balance isolating more bacteria with reducing the workload. In this study, two aspects of the bacterial isolation strategy were investigated: the number of colonies to select from each plate, and the optimal sampling time-points over the course of the incubation period. In fact, we had adopted the “picking all” at all time points in sample F1, and it was found that the number of bacteria in these two time-points was the largest, so the two time points were selected. Meanwhile, in order to meet statistical analysis, three samples were selected. Regarding colony selection, we found that 32.9% of bacteria were missed by the experienced picking method compared to the picking all method at a single time-point, but as the pre-incubation time progressed, only 8.5% of the bacteria were missed. Therefore, at later time-points in the pre-incubation period, picking colonies by the experienced picking method is practicable. Compared with all selection, experienced picking can reduce the workload by at least 85%. Regarding optimization of the sampling time-points, we compared the number of bacterial species identified from each sample at each time-point, and under anaerobic (0, 3, 6, 9, 15, 27, and 30 days) and aerobic (0, 3, 6, 9, 15, and 27 days) culturing conditions to select the optimal combination. Using the optimal combination of factors, more than 90% of the bacteria identified using the original method were isolated, and the workload was reduced by nearly 40%. This also indicates that the same amount of work resulted in the isolation of more species using the improved method compared with the original method.

Currently, bacteria whose 16S rRNA gene sequences have less than 98.65% similarity compared with those of known species are considered to be potentially new species. Based on this criterion, we found three novel species in the human gut. Two of them were isolated from the supplemented group, which suggests that the conditions in this group were probably more conducive to the isolation of unknown bacteria compared with the non-supplemented group. In addition, these three species were isolated from samples taken on day 9, 15, and 30, indicating the usefulness of taking pre-cultures at these optimized time points.

This method provides us with a better strategy for picking bacteria. Under the premise of isolating as many bacteria as possible, our work load is reduced, however, there are some limitations. For example, in order to avoid cross-contamination between samples, it needs to be cultured independently, if there are more samples, it will inevitably increase the time of the experiment. In addition, only one YCFA medium was used in this experiment, and YCFA medium could not be suitable for the growth of all bacteria, so the application of various media for culture will be a direction of our future research.

Overall, supplementing the culture with fresh medium yielded more bacteria than the non-supplemented group. The optimized pre-incubation time-points and the experienced picking method significantly decreased the workload required for this approach. Three novel species were identified, and eight species were isolated from the gut or human body for the first time, thus improving our knowledge of novel bacteria in the gut. We have applied this strategy to other culture conditions, such as the application of inhibitors, and oligotrophic culture. We also used this method to isolate bacteria from colorectal cancer mucosa tissues. Thus, this newly optimized culturomics strategy will be useful for obtaining more bacteria with less work in future studies of the gut microbiota.

## Data Availability Statement

All datasets generated for this study are included in the article/Supplementary Material.

## Ethics Statement

The research was approved by the Ethics Committee of Beijing Shijitan Hospital (Beijing, China; agreement no. 2018KY55). The patients/participants provided their written informed consent to participate in this study. Written informed consent was obtained from the individual(s) for the publication of any potentially identifiable images or data included in the manuscript.

## Author Contributions

YC did the experiments, analyzed the data, and wrote the manuscript. FH did the experiments and analyzed the data. ZP, ZH, and NH analyzed the data. LB, HD, and ZL investigated the literature. LD, HG, and FZ collected and pretreated the samples. RY directed the experiments and contributed to revising the manuscript. YB designed the experiments, provided overall directions, and contributed to revising the manuscript.

## Conflict of Interest

The authors declare that the research was conducted in the absence of any commercial or financial relationships that could be construed as a potential conflict of interest.

## Supplementary Material

FIGURE S1 **(A)** Genus composition of each stool sample. **(B)** Numbers of bacterial species found in sample(s).

FIGURE S2 **(A)** Bacteria isolated from fecal sample 1 over time. **(B)** Bacteria isolated from fecal sample 2 over time. **(C)** Bacteria isolated from fecal sample 3 over time. **(D)** Bacteria isolated from fecal sample 5 over time. The bacteria identified at each time point are represented by horizontal lines of different colors. ^∗^indicates bacteria that were only isolated from the supplemented group, and ^#^indicates bacteria that were only isolated from the non-supplemented group. The color scale at the bottom of the figure indicates the number of bacterial species isolated from the sample taken at each time point.

FIGURE S3 **(A)** The number of bacteria varies with time under aerobic conditions. **(B)** The number of bacteria varies with time under anaerobics.

TABLE S1 The numbers of colonies analyzed for each condition.
