# Supplementary figures and images for: Optimization of Culturomics Strategy in Human Fecal Samples
Source: Front Microbiol. 2019 Dec 17;10:2891. doi: 10.3389/fmicb.2019.02891 (PMC6927924; doi:10.3389/fmicb.2019.02891)

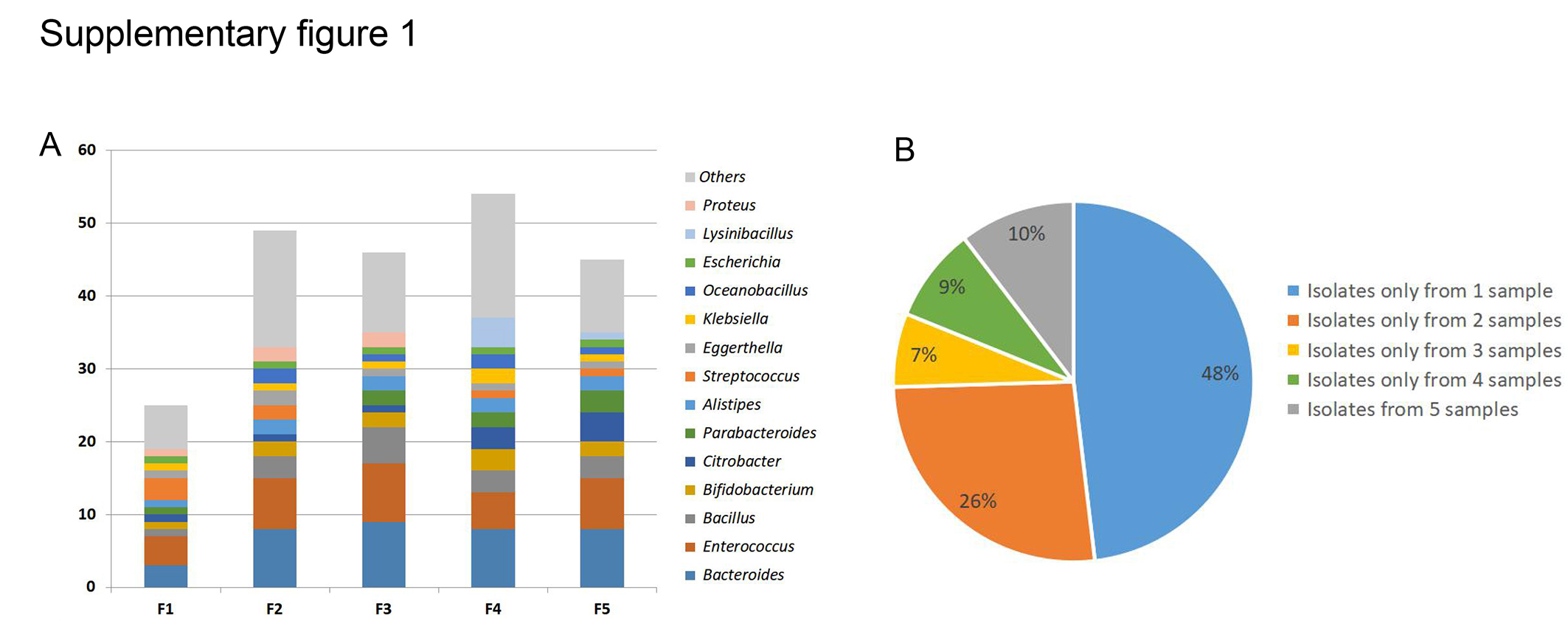

Supplement: FIGURE S1 — (A) Genus composition of each stool sample. (B) Numbers of bacterial species found in sample(s). [file Data_Sheet_1.zip › Supplementary Figure 1.JPEG]

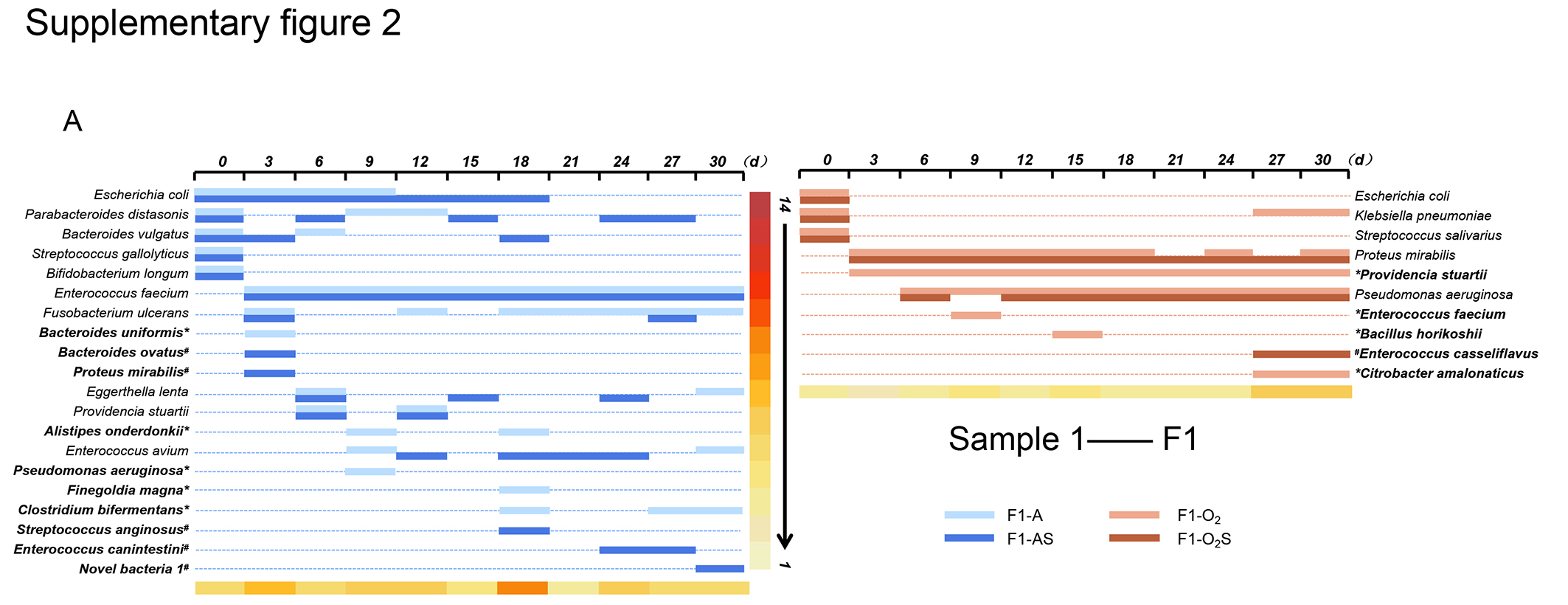

Supplement: FIGURE S1 — (A) Genus composition of each stool sample. (B) Numbers of bacterial species found in sample(s). [file Data_Sheet_1.zip › Supplementary Figure 2A.JPEG]

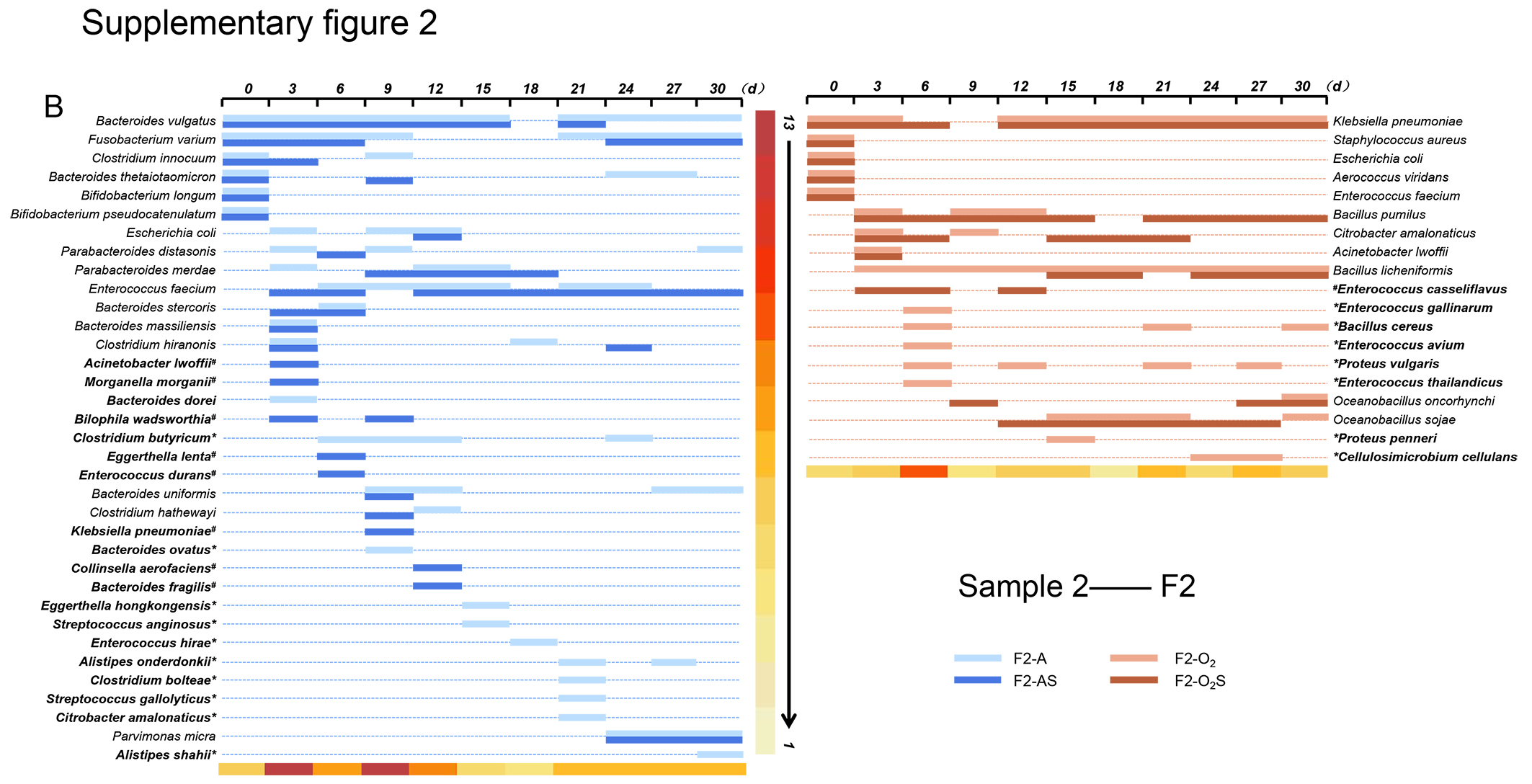

Supplement: FIGURE S1 — (A) Genus composition of each stool sample. (B) Numbers of bacterial species found in sample(s). [file Data_Sheet_1.zip › Supplementary Figure 2B.JPEG]

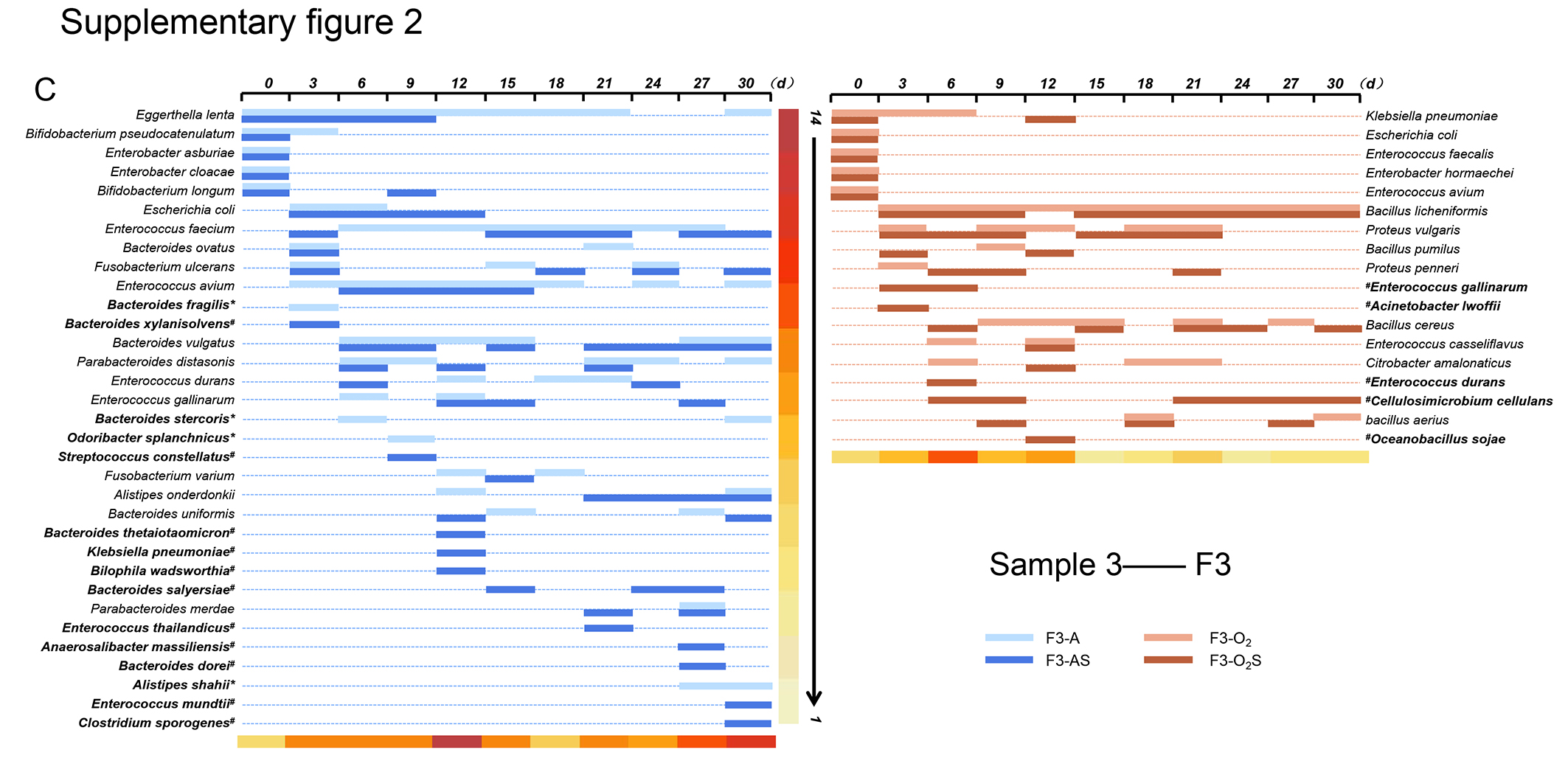

Supplement: FIGURE S1 — (A) Genus composition of each stool sample. (B) Numbers of bacterial species found in sample(s). [file Data_Sheet_1.zip › Supplementary Figure 2C.JPEG]

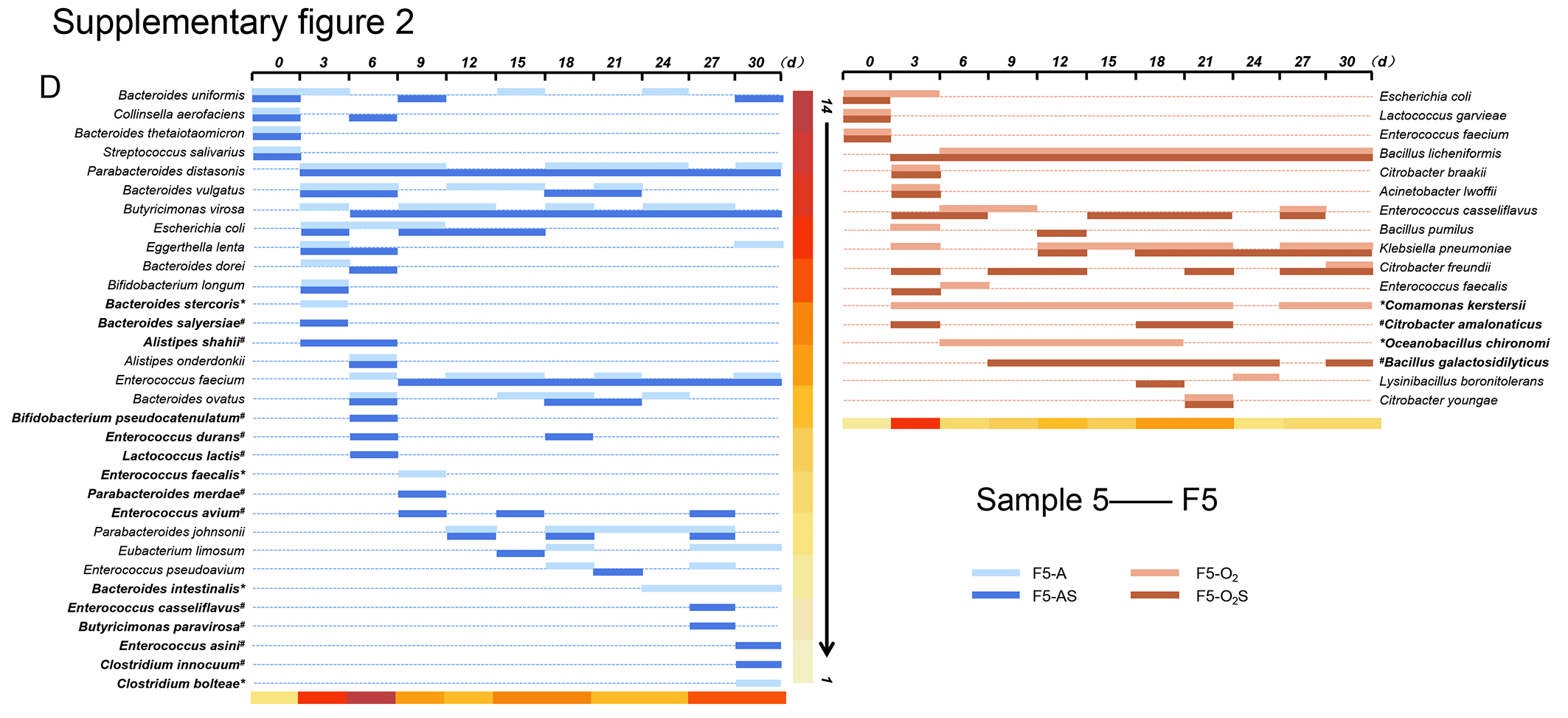

Supplement: FIGURE S1 — (A) Genus composition of each stool sample. (B) Numbers of bacterial species found in sample(s). [file Data_Sheet_1.zip › Supplementary Figure 2D.JPEG]

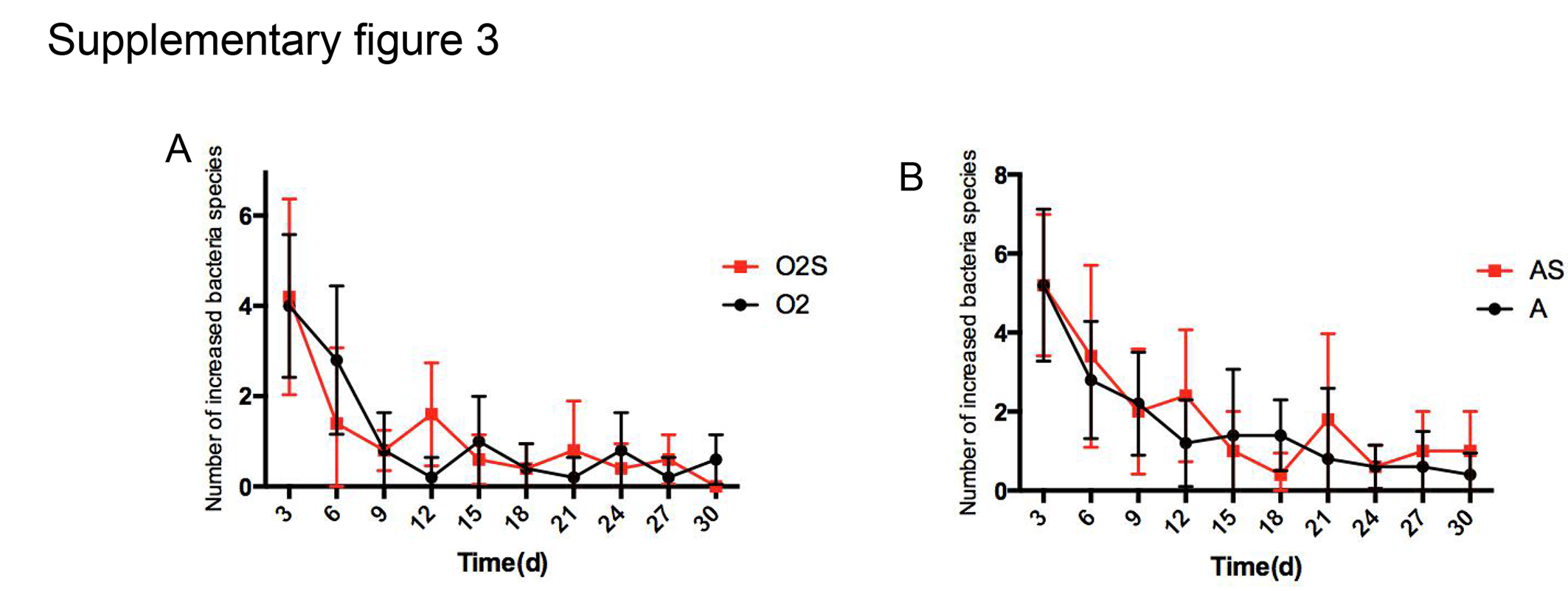

Supplement: FIGURE S1 — (A) Genus composition of each stool sample. (B) Numbers of bacterial species found in sample(s). [file Data_Sheet_1.zip › Supplementary Figure 3.JPEG]
